# The Influence of an Isocyanate Structure on a Polyurethane Delivery System for 2′-Deoxycytidine-5′-monophosphate

**DOI:** 10.3390/jfb14100526

**Published:** 2023-10-18

**Authors:** Florin Borcan, Titus Vlase, Gabriela Vlase, Roxana Popescu, Codruta M. Soica

**Affiliations:** 1 Department I, Advanced Instrumental Screening Center, Faculty of Pharmacy, “Victor Babes” University of Medicine and Pharmacy, 2 E. Murgu Sq., 300041 Timisoara, Romania; 2 Research Center “Thermal Analysis in Environmental Problems”, Faculty of Chemistry, Biology, Geography, West University of Timisoara, 16 Pestalozzi Str., 300115 Timisoara, Romania; titus.vlase@e-uvt.ro (T.V.); gabriela.vlase@e-uvt.ro (G.V.); 3 Department II, Faculty of Medicine, “Victor Babes” University of Medicine and Pharmacy, 14A T. Vladimirescu Str., 300041 Timisoara, Romania; popescu.roxana@umft.ro; 4 Department II, Faculty of Pharmacy, “Victor Babes” University of Medicine and Pharmacy, 2 E. Murgu Sq., 300041 Timisoara, Romania; codrutasoica@umft.ro

**Keywords:** cell cultures, drug delivery, FT-IR, mice skin, thermal analysis, UV-Vis, Zetasizer

## Abstract

The delivery of nucleosides represents an interesting research trend in recent years due to their application in various viral infections. The main aims of this study were to develop and to characterize polyurethane particles that are intended to be used for the transport of nucleosides. Three samples have been prepared using aliphatic diisocyanates, a mixture of polyethylene glycol, polycaprolactone, and diols, respectively. The samples were characterized through refractivity measurements, drug loading efficacy, release and penetration rate investigations, FTIR and Raman spectroscopy, thermal analyses, Zetasizer, SEM, HDFa cells viability, and irritation tests on mice skin. The results indicate the obtaining of particles with sizes between 132 and 190 nm, positive Zeta potential values (28.3–31.5 mV), and a refractivity index around 1.60. A good thermal stability was found, and SEM images show a medium tendency to agglomerate. The samples’ color, pH, and electrical conductivity have changed only to a small extent over time, and the evaluations indicate an almost 70% encapsulation efficacy, a prolonged release, and that around 70% of particles have penetrated an artificial membrane in the first 24 h. The synthesized products should be tested in further clinical trials, and the current tests on cell cultures and mice skin revealed no side effects.

## 1. Introduction

**Figure 1:**
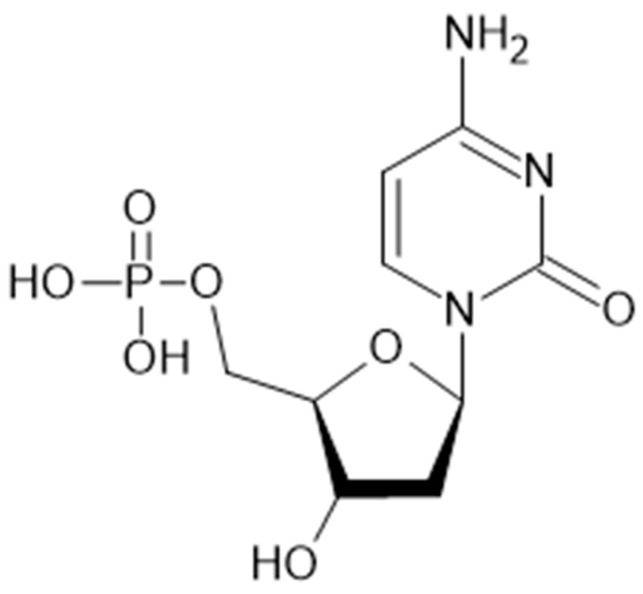
The chemical structure of dCMP.

Nucleosides represent a large group of biological molecules known as glycosylamines, displaying similar structures to nucleotide structures but lacking phosphate functional groups. They exhibit very diverse structures containing a nucleobase or nitrogenous base (adenine, thymine, cytosine, guanine) that is covalently linked to a monosaccharide with five carbon atoms [1,2]. The nucleosides act as precursors in the synthesis of nucleic acids, and they are also involved in the control of the metabolism and growth of all living cells; additionally, they modulate certain activities of the muscular, cardiovascular, and nervous systems [3]. Various supplements based on exogenous nucleosides (and nucleotides) have been developed for patients who lack the ability to synthesize these compounds de novo [4]. The challenges and advantages associated with the use of drug delivery systems for nucleosides can also be associated with various approaches in cancer treatment [5,6]. 2′-Deoxycytidine-5′-monophosphate (dCMP, Figure 1), often found as a substrate of uridine monophosphate, is a compound that contains cytosine (2-oxy-4-amino-pyrimidine) as the nitrogenous base linked to a deoxyribose and a phosphate group; in the DNA double helix, dCMP is paired with deoxyguanosine-monophosphate (dGMP) [7]. The reactivity in water, the functionalization, and the encapsulation of its salts were highly investigated in the last two decades to enhance its bioavailability [8,9]. The analogues of dCMP, such as 5-AZA-dCMP [10] or its conjugate with delta-di-carboxylic amino acid [11], are highly investigated as substrates for deamination and DNA polymerization. Despite the large number of nucleotides analogues that have already been investigated and approved in therapy, the lack of studies on their encapsulation causes this research field to be very interesting.

Polyurethanes (PUs) are polymer compounds that contain urethane/carbamate groups (-NH-CO-O-), originated from the reaction between (bi- or multi-)functional isocyanates and (bi- or multi-)functional compounds with active hydrogen like alcohols and diamines. They were discovered almost 90 years ago, and they are found in various forms in many applications due to the ease of final properties modification and their versatility and biocompatibility [12]. PU drug delivery systems consist of a small group of nano- and microparticles, capsules, and structures that have been developed since the start of the last decade of the last century. Although this polymer class was initially developed industrially (as foams, adhesives, and coatings), in the second part of the last century, various biomedical applications began to appear due to the versatility, lack of toxicity, and hemocompatibility of the finished products [13,14,15]. The first syntheses of polyurethane carriers were based on aromatic compounds (methylene-diphenyl diisocyanate or MDI and toluene diisocyanate or TDI) and resulted in large structures with low penetration rates; hence, it was necessary to find a synthesis method that was able to reduce the carrier size. Several studies by K. Bouchemal et al. [16,17,18] describe colloidal suspensions of polyurethane capsules with much smaller sizes than their predecessors; their synthesis, based on an interfacial polyaddition process combined with a spontaneous emulsification, used a mixture of polyethylene glycol, ethylene glycol, butanediol, and hexanediol as the aqueous phase, isophorone diisocyanate, and two surfactants (a hydrophilic one, Tween^®^ 20, and a lipophilic one, Span^®^ 85). The mechanism of the transmembrane transport via polymer delivery systems depends on the physical and chemical properties of their particles, such as size, morphology, surface charge, etc. To date, there has been relatively few investigations on the mechanism of PU particles. Y. Niu et al. [19] have described that PU nanoparticles flanked by polyethylene glycol and polycaprolactone segments have the ability to undergo endocytic uptake, followed by endosomal escape.

The investigation of the nucleosides’ potential and the development of new analogues began in 1960s [20]. Many analogues based on different changes of the sugar scaffold (at the 2′- and/or 3′-OH group) or of the oxygen from the furanose ring are already approved as antiviral agents. Different nucleoside delivery systems have also been developed in recent decades, but a few difficulties encountered in the nucleosides-based therapies lie in hepatic injury, bone marrow suppression, pancreatitis, etc. [21]. Polyurethane carriers represent an attractive option due to their easy-to-control extended release that has already been described in the literature [22].

Our research team has synthesized and characterized different polyurethane structures that can be used as carriers for various extracts [23,24,25] and drugs [26,27] in the last 15 years; the recipe of the synthesis and the raw materials have been continuously modified, ensuring that the size of the particles allows for the most efficient encapsulation and an easy-to-control release time. On the other hand, we managed to reduce the number of precursors by eliminating one of the surfactants and the catalyst, thus reducing the toxicity potential while maintaining optimal surface charge values in order to prevent particle agglomeration. To the best of our knowledge, the presence of polyurethanes as carriers for nucleosides and nucleic acids is very limited; therefore, the main aim of the current study was to obtain and characterize an efficient and safe polyurethane drug delivery system suitable for the transmembrane transfer of nucleosides and to compare the properties of the final synthesized products based on isocyanates with different chemical structures.

## 2. Materials and Methods

### 2.1. Materials and Animals

Tween^®^ 20 and hexamethylene diisocyanate (HDI) were purchased from Merck (Darmstadt, Germany), isophorone diisocyanate (IPDI), polyethylene glycol (PEG 400), polycaprolactone diol (PCL Mn~530), 1,6-hexanediol (HD), tris(hydroxymethyl)amino-methane (THAM), inorganic salts (NaCl, KCl and MgCl_2_, Na_2_HPO_4_, K_2_HPO_4_, KH_2_PO_4_, and NaHCO_3_), and 2′-Deoxycytidine 5′-monophosphate as sodium salt were acquired from Sigma Aldrich (St. Louis, MO, USA). Lysine diisocyanate (LDI) was obtained from Imaginechem Co. (Hangzhou, China), and acetone from Honeywell Riedel-de Haen (Seelze, Germany). All the reagents were analytical-grade purity, and they were used without any previous purification; double-distilled water was prepared in-house using a JP Selecta Dest-4 distiller.

For the in vitro experiments using cell cultures, HDFa (Human Dermal Fibroblast) was purchased from Invitrogen (Waltham, MA, USA), while the reagents (culture media, fetal bovine serum (FBS), antibiotics, phosphate-buffered saline (PBS), 0.25% trypsin-EDTA solution, MTT solution, dimethylsulfoxide) and culture supplies (culture flasks, Pasteur pipettes, pipette tips, conical tubes, 96-well microplates) were obtained from Thermo Fischer Scientific (Waltham, MA, USA).

Male, 7–9-week-old CB17SCID and Nu/Nu, Balb-c mice were purchased from Charles River (Sulzfeld, Germany). Mice were kept under standard conditions—temperature (23 ± 2 °C), humidity (50–60%), 12 h light/dark cycle; they were fed ad libitum.

The study was conducted in accordance with the Declaration of Helsinki and approved by the Ethics Committee of “Victor Babes” University of Medicine and Pharmacy Timisoara (approval no. 42 from 29 October 2021).

### 2.2. Synthesis of the PU Carriers

**Figure 2:**
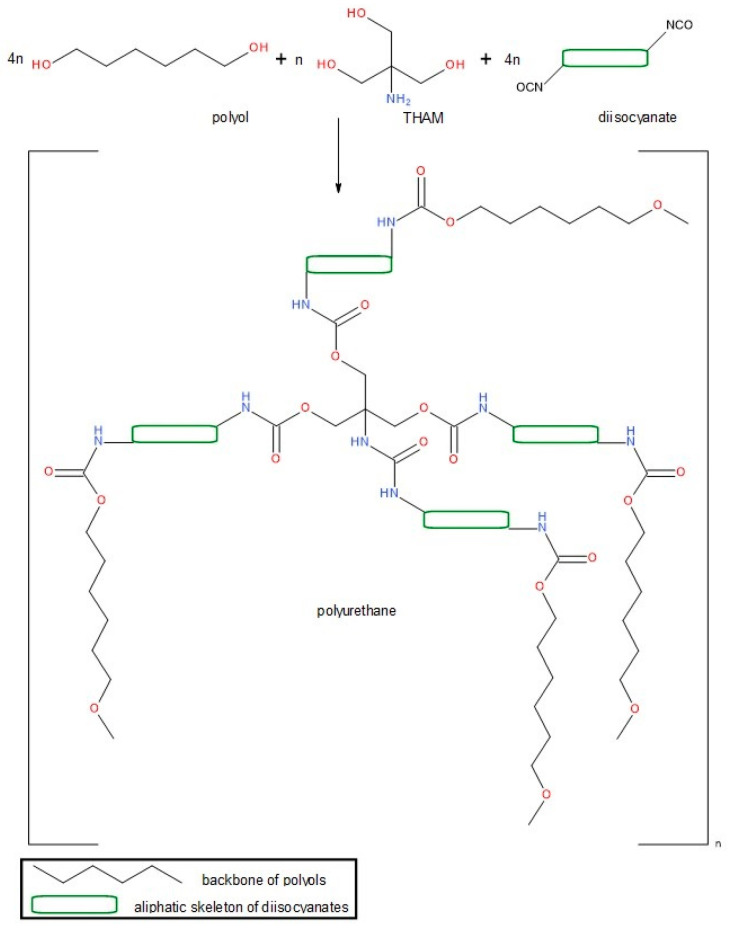
Schematic illustration of the chemical reaction.

The synthesis of the drug delivery system is based on an exothermic polyaddition reaction between an active hydrogen-containing phase (mixture of different ether and/or ester polyols) and a diisocyanate (Figure 2). In this study, aliphatic isocyanates (hexamethylene diisocyanate, isophorone diisocyanate, and lysine diisocyanate) have been chosen in order to avoid the carcinogenic risk of aromatic compounds. The mixture of polyols (PEG 400 and PCL) and their ratio were established according to our previous results [28,29], taking into consideration that the balance between the ether and ester polyols has a major impact on the drug release kinetics.

The active hydrogen-containing phase (a mixture of 1.75 mL HD, 3.50 g PEG 400, 3.10 g PCL, 1.15 g THAM, and 0.50 g 2′-Deoxycytidine 5′-monophosphate sodium salt) and 90 mL of double-distilled water were added to a Duran^®^ beaker and stirred on a Velp hot plate stirrer (Usmate, Italy) at 350 rpm and 35 ± 0.2 °C overnight. In parallel, three isocyanate solutions obtained from 6.00 mL HDI, IPDI, and LDI, respectively, in 50.00 mL acetone, were homogenized in three cone-shaped flasks at 350 rpm and 40 ± 0.2 °C for 30 min. Then, the content of the beaker was divided equally among the three flasks and their contents continued to be stirred at 350 rpm and 40 ± 0.2 °C for 3 h to complete the synthesis of the macromolecular chains; the temperatures were precisely monitored using a P700 Universal thermometer from Dostmann electronic GmbH (Wertheim, Germany). In the end, the contents of the three flasks were sonicated for 5 min using a Bandelin Sonorex Digitec bath sonicator (Berlin, Germany). The three samples were labeled as follows: str 1 (based on HDI), str 2 (based on IPDI), and str 3 (based on LDI).

**Figure 3:**
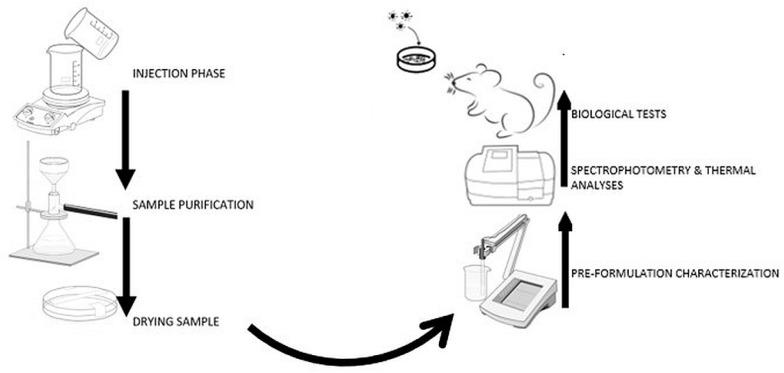
The scheme of synthesis and characterization of samples.

The obtained suspensions were repeatedly washed with a mixture of water:acetone (1:1.4, *v*/*v*), were passed through 0.22 μm PVDF sterile syringe filters from Merck Millipore (Darmstadt, Germany), and then centrifuged at 2000× *g* using an Ika Mini G microcentrifuge (Staufen, Germany) in order to concentrate the samples; the mixture used to wash the samples was later analyzed in order to establish the free drug content. Finally, the samples were dried in borosilicate glass Petri dishes at 45 °C inside a PolEko SL115 drying oven to constant mass (almost 30 h). The synthesis steps and the characterization of samples are shown in Figure 3.

### 2.3. Preformulation Characteristics

The sample pH was evaluated using a Mettler Toledo FiveGo F2 portable pH meter (Schwerzenbach, Switzerland), InLab^®^ Expert Go Sensor, and aqueous solutions with the same concentration (0.8 mg mL^−1^) at the same temperature (25 °C). The instrument was previously calibrated using four different buffer solutions (pH = 4.01, 7, 9.21, and 11), provided by the same manufacturer.

The refractive index of the synthesized samples was obtained using 2–3 drops of every aqueous solution on a Kern digital refractometer (Balingen, Germany) at room temperature.

A series of dissolution tests were conducted in distilled water, acetone, and DMSO, respectively, following a procedure already described in the literature [23]: 5.0 mg dried powder was dissolved in the respective solvent in 20 mL glass vials with screw caps at room temperature; the volume of every solvent was registered when a single, clear liquid phase with no distinct solid or gel particles was recorded.

### 2.4. Drug Loading

The samples were dissolved in distilled water (0.8 mg mL^−1^) to determine the drug loading efficacy, following a procedure described in the literature [30]. The amount of 2’-Deoxycytidine-5’-monophosphate sodium salt successfully entrapped inside the carrier particles was found by reporting the quantity of the free drug to the total added amount. The amounts were calculated using the Beer–Lambert law at 271 nm and an SI Analytics UVi Line 9400 Spectrophotometer (Mainz, Germany); a calibration curve was initially drawn by plotting the absorption values as a function of drug concentration, and it can be described by Equation (1):

y = 0.1261 x, R^2^ = 0.9829 (1)

where: y = absorption value, x = drug concentration (µg/mL), and R^2^ = the coefficient of determination.

### 2.5. Release Profile

The cumulative drug release (CDR) was calculated by measuring the amount of the active agent that was released during the carrier’s exposure to simulated body fluid (SBF), prepared according to F. Baino and S. Yamaguchi [31]; SBF is often used for in vitro measurements on artificial biomaterials under biomimetic conditions. First, the solution of SBF was prepared as follows: first, 6.55 g NaCl, 2.27 g NaHCO_3_, 0.37 g KCl, and 0.14 g Na_2_HPO_4_ were mixed with 960 mL double-distilled water in a 1000 mL capacity flat bottom flask and heated at 37 °C; then, 0.31 g MgCl_2_ hexahydrate, 0.37 g CaCl_2_ dihydrate, 0.07 g Na_2_SO_4_, and 6.06 g Tris were added, and the pH was adjusted to 7.4 with a solution HCl 1M. The samples were introduced into the degrading environment (3.0 mg sample in 25.0 mL SBF) and kept for 5 days at 37 °C and 150 rpm; at specific time points (daily), 1.0 mL of SBF was replaced with fresh degrading environment, and UV-Vis spectroscopy was employed to quantify the released drug. The cumulative percentage of drug release was calculated based on the ratio between the difference of total dCMP amount and the released quantity vs. the total amount of dCMP added during the synthesis [32].

### 2.6. The Penetrability Rate

A small in vitro Franz-type vertical static diffusion cell system (Φ 15 mm, diffusion area of 1.77 cm^2^, and a receptor volume of 12.0 mL) and a PVDF artificial membrane Spectra/Por^®^ were used to test the carrier permeation. The receptor compartment was filled with phosphate-buffered saline in such a way that air bubbles would not remain between the artificial membrane and the receptor fluid. The experiment was conducted at 25 ± 1 °C, and at every 10 h, 1.0 mL liquid from the receptor compartment was replaced with fresh buffer and spectrophotometrically analyzed between 356 and 362 nm depending on the sample/diisocyanate type.

### 2.7. FTIR-UATR Spectra

FTIR-UATR analysis was performed using a Shimadzu AIM-9000 device by employing the attenuated total reflectance (ATR) technique without a preliminary preparation of the dried samples. The data were collected after 20 consecutive readings at a resolution of 4 cm^−1^, within the 4000–280 cm^−1^ range.

### 2.8. Raman Spectroscopy

The spectra were collected with a LabRAM Soleil V1.0—Confocal Raman Microscope (Horiba Scientific, Kyoto, Japan): the dried samples were compressed with a hydraulic press to obtain circular discs, and a 785 nm (red) laser with a deviation angle (DV) and a diffraction grating of 500 nm was used. The spectra were collected from Raman shift 30 to 2000 cm^−1^ using a 5× objective lens; each spectrum was obtained using an acq. time of 30 s and two accumulations.

### 2.9. Thermal Analysis

The thermal stability of samples was investigated using a Thermal Analyzer TGA/DSC3+ STARe System (Mettler Toledo, Port Melbourne, Australia). Analyses were performed between 25–500 °C in a dynamic air atmosphere (100 mL/min, synthetic air) with a 10°/min heating rate. Small amounts of every sample (between 3.5 and 4.5 mg) were placed in 40 μL aluminum melting crucibles with a pin and sealed with pierced lids using a DSC-204 Netzsch differential scanning calorimeter (Selb, Germany) under the same conditions (atmosphere, heating rate, and temperature range).

### 2.10. Zetasizer Tests

The size and surface charge of samples were evaluated through dynamic light scattering technique on a Cordouan Technol. system (Pessac, France) containing a nanosized detector (Vasco analyzer) and a charge detection module (Wallis). The following input parameters were set for the determination of particle size and distribution: temperature (22 °C), time interval (18 μs), channels number (460), laser power (85%), acquisition mode (continuous), and analysis mode (Pade–Laplace). The following parameters were set to detect the surface charge: temperature (22 °C), applied field (automatic), resolution (0.8 Hz—medium), measures number/sequence (3), laser power (75%), Henry function (Smoluchowski), and cuvettes (quartz with applicability between 190 and 2500 nm).

### 2.11. SEM Analysis

Sample morphology was assessed using a compact and versatile JSM-IT200 scanning electron microscope (Peabody, MA, USA). The following parameters were set: landing voltage (10.0 kV), WD (9.6 mm), magnification (1400×), scan rotation (336.7°), and pressure (40 Pa).

### 2.12. Sample Stability

The stability of PU carriers was evaluated as previously described [16]: briefly, an aliquot of every sample was maintained at 3 different temperatures (respectively, 8 ± 0.5 °C in a refrigerator, 25 ± 0.5, and 40 ± 0.5 °C with 65 ± 3% relative humidity in a laboratory incubator) for 30 days; various parameters such as color, electrical conductivity, and pH were recorded every third day. The color stability was evaluated using the changes in A_max_ and an SI Analytics UVi Line 9400 spectrophotometer (Mainz, Germany) according to a modified procedure described in the literature [22]: every sample was dissolved in water every third day and the absorptivities were read in triplicate at two wavelengths (350 and 540 nm), and they were reported to the values initially recorded; the double-distilled water was used as reference to validate the spectrophotometric results. The electrical conductivity was assessed using a Jenway Bench 4010 Conductivity meter (Staffordshire, UK) at 25 °C in aqueous solutions (0.8 mg mL^−1^).

### 2.13. Cell Viability

HDFa cells (passage 3) were used for the experiment, with the cells being seeded in a medium supplemented with 10% fetal bovine serum and 1% penicillin-streptomycin. Cells were grown under standard conditions in an incubator in the specific environment already presented. The medium was changed regularly every 2–3 days. The proliferation of cells was determined via the MTT colorimetric technique. For the biocompatibility analysis of the compounds, cells from the HDFa line (2 × 10^4^ cells/well) were seeded in 100 µL medium in 96-well plates. Cells were allowed to adhere for 24 h in the incubator. After 24 h, the growth medium was changed with the test compounds, diluted in the medium at different ratios: 90 µL medium + 10 µL sample, 95 µL medium + 5 µL sample, and 99 µL medium + 1 µL sample (0.080 mg mL^−1^, 0.040 mg mL^−1^, and 0.008 mg mL^−1^). In parallel, we used control batches of cells treated with the DMSO, in similar concentrations to those in the test samples. The tested samples were suspended in DMSO. All tests were performed in triplicate. At 24, 48, and 72 h after the treatment, the cell proliferation tests were performed using the MTT assay, and the absorbance at 590 nm was measured using a Bio-Tek Synergy H1 spectrophotometer (Santa Clara, CA, USA).

The percentage of cell proliferation is described by Equation (2) [33]:

Proliferation (% control) = (At/Ac) × 100(2)

where At is the absorbance for the test solutions, and Ac is the absorbance for the control. The average values for three consecutive determinations were used for the calculation.

### 2.14. Skin Irritation Evaluations

A pharmaco-toxicological evaluation for the newly synthesized products was performed using sensitive mice with and without hair. The mice were divided into five groups, labeled as the three PU carrier samples and two special groups (control group—mice treated only with the solvent, and SLS group—mice treated with a 2.0% sodium lauryl sulfate solution, a recognized skin irritant). The hair from the back of CB17SCID mice (3–4 cm^2^) was shaved at the beginning of experiment and then every 5 days. The application of the studied samples (40 ± 3 µL once) was performed on the back skin every third day for 15 days, and the measurements of parameters were performed 30 min later by the same operator under the same conditions (temperature and humidity). The modification of skin erythema was monitored using a Mexameter^®^ MX 18 probe, while the skin hydration was evaluated using a Corneometer^®^ CM 825 probe, both of them being connected to a Multiprobe Adapter System (MPA5) from Courage-Khazaka (Koln, Germany).

### 2.15. Statistics

Data analyses were performed using IBM SPSS Software version 27.0.0.0 (Armonk, NY, USA). The continuous variables are presented as mean and standard error (SE). The dataset normality was assessed using the Kolmogorov–Smirnov (K-S) test followed by the ANOVA and Kruskal–Wallis tests. *p* < 0.05 was considered statistically significant. The charts were modeled in Excel—Microsoft^®^ Office Professional Plus 2019 from Microsoft Corp. (Redmond, WA, USA).

## 3. Results

### 3.1. Preformulation Characteristics

The evaluation of pH reveals the neutral and safe character of the samples. The following results were obtained: 6.69 ± 0.16 (sample str 1), 6.83 ± 0.12 (str 2), and 6.78 ± 0.08 (str 3). All samples were found to have pH values that fall within the acceptable range for pharmaceutical products that are administered via the oral route [34].

The values of the refractivity index were 1.59 for str 1 and str 3 samples, and 1.61 for the str 2 sample. An increased value for the sample based on an aliphatic and cyclic diisocyanate (IPDI) was obtained, while the use of the strain-chain isocyanates (HDI and LDI) as raw materials leads to PU carriers with decreased refractivity index values. These values are very important for the characterization of samples, because the analysis can be used to verify the purity and the isotropic nature of the samples.

Table 1 presents the solubility of samples in different solvents.

Small differences were found between the solubility of the three samples in three different solvents; the samples based on straight-chain diisocyanates present a higher solubility in water and DMSO, while the str 2 sample, based on a cyclic isocyanate, shows increased values in acetone.

### 3.2. Drug Loading

The difference between the maximum absorbances of the carrier and the loaded active substance was used to determine the amount of free active substance; a graph of the absorbance vs. calibration was drawn based on the absorbance of four standard solutions of different concentrations. Average encapsulation efficiencies equal to 67.2 ± 4.1% (str 1), 68.9 ± 3.7% (str 2), and 68.1 ± 4.4% (str 3) were obtained by reporting the amount of free drug to the total quantity added to the synthesis.

### 3.3. The Release Profile

**Figure 4:**
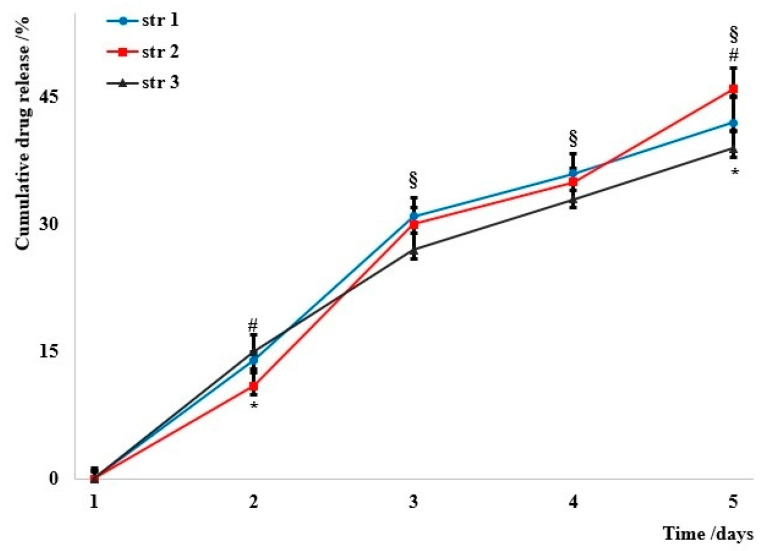
The evolution of the cumulated drug release. Significant differences *p* < 0.05, # (str 1 vs. str 2), § (str 1 vs. str 3), * (str 2 vs. str 3).

The cumulated drug release is presented in Figure 4; data were collected in nearly perfect sink conditions that were described in a previous study [35].

### 3.4. Penetrability Rate

**Figure 5:**
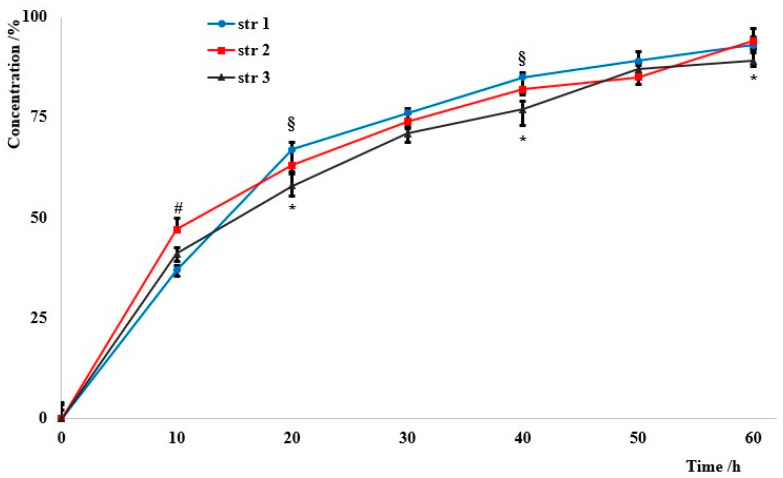
The penetrability rates. Significant differences *p* < 0.05, # (str 1 vs. str 2), § (str 1 vs. str 3), * (str 2 vs. str 3).

The penetrability through various membranes is an important rate-limiting parameter that influences the release of the active agent to the target. The permeation through an artificial membrane was quite similar for all samples (Figure 5): almost 50% of particles penetrated in the first ten hours, and around 70% passed through the membrane in the first 24 h.

### 3.5. FTIR-UATR

**Figure 6:**
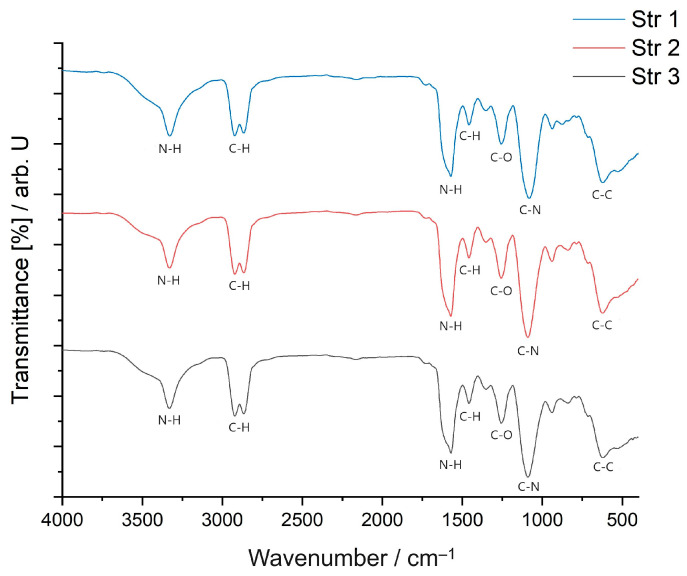
FTIR spectra of samples.

Figure 6 displays the FTIR spectra of the analyzed samples.

Comparatively, the FTIR-ATR spectra reveal many similarities between the synthesized samples. The spectra of the sample based on HDI (str 1) shows an absorption band at 3331 cm^−1^, corresponding to -NH- stretching. The two sharp bands at 2855 cm^−1^ and 2924 cm^−1^, respectively, are associated with -CH_2_- stretching, while displacements of the -CH_2_- bond are indicated by the bands at 1460, 1440, 1353, and 1278 cm^−1^. In addition, the absorption band specific to the urethane C=O bond occurs at 1734 cm^−1^. The displacements of the -NH- bond appear at 1617 and 1577 cm^−1^. The C-O-C bond can be identified by the presence of the bands at 1093 and 1075 cm^−1^. There are also characteristic bands of primary or secondary aliphatic alcohols in the range of 1480–1405 cm^−1^ and 1075–1000 cm^−1^. Mono-substituted alkyl ether-type structures are indicated by the presence of bands in the range of 1515–1455 cm^−1^, 1260–1235 cm^−1^, 1080–980 cm^−1^, and 755–730 cm^−1^.

The second sample (str 2) shows an absorption band, as in the case of the previous sample, at 3331 cm^−1^ corresponding to the -NH- stretch; two intense bands that can be attributed to the -CH_2_- stretching occur at 2855 cm^−1^ and 2926 cm^−1^, while the displacements of the -CH_2_- bond are identified by 1460, 1438, 1352, and 1278 cm^−1^ bands. The absorption band specific to the urethane C=O bond is present at 1734 cm^−1^ and at 1670 cm^−1^. The displacements of the -NH- bond appear at 1617 and 1577 cm^−1^, respectively. The C-O-C bond is indicated by the presence of the bands at 1093 and 1075 cm^−1^. There are also bands characteristic of primary or secondary aliphatic alcohols in the range of 1480–1405 cm^−1^ and 1075–1000 cm^−1^. Structures of the aliphatic cyclic ether-type can be identified by the bands within the 1400–1350 cm^−1^, 1135–1050 cm^−1^, 990–940 cm^−1^, and 880–830 cm^−1^ ranges.

The FTIR spectra of the str 3 sample contains an absorption band corresponding to the -NH- stretching at 3328 cm^−1^ and two intense bands that can be attributed to -CH_2_- stretching at 2855 cm^−1^ and 2926 cm^−1^, while the displacements of the -CH_2_- bond are identified at 1460, 1438, 1352, and 1278 cm^−1^. In addition, two absorption bands specific to the urethane C=O bond are present at 1734 cm^−1^ and at 1670 cm^−1^, respectively, while the displacements of the -NH- bond appear at 1616 and 1576 cm^−1^. The C-O-C bond is indicated by the presence of bands at 1090 and 1073 cm^−1^. There are also bands characteristic of primary or secondary aliphatic alcohols in the range of 1480–1405 cm^−1^ and 1075–1000 cm^−1^. Structures of the aliphatic cyclic ether-type can be identified by the occurrence of bands within the 1400–1350 cm^−1^, 1135–1050 cm^−1^, 990–940 cm^−1^, and 880–830 cm^−1^ ranges.

No obvious difference was observed between these three samples and by comparing them with other data on PU materials [36]. The spectrum of the active substance has already been published [37]; it shows a scissoring band of amino groups at 1717 cm^−1^, pyrimidine ring vibrations between 1600 and 1529 cm^−1^, a C-N stretch at 1491 cm^−1^, and three broad bands specific to the phosphate group at 1085, 1002, and 948 cm^−1^, where the first two represent the antisymmetric and symmetric stretch of PO_2_^−^, respectively.

### 3.6. Raman Spectroscopy

**Figure 7:**
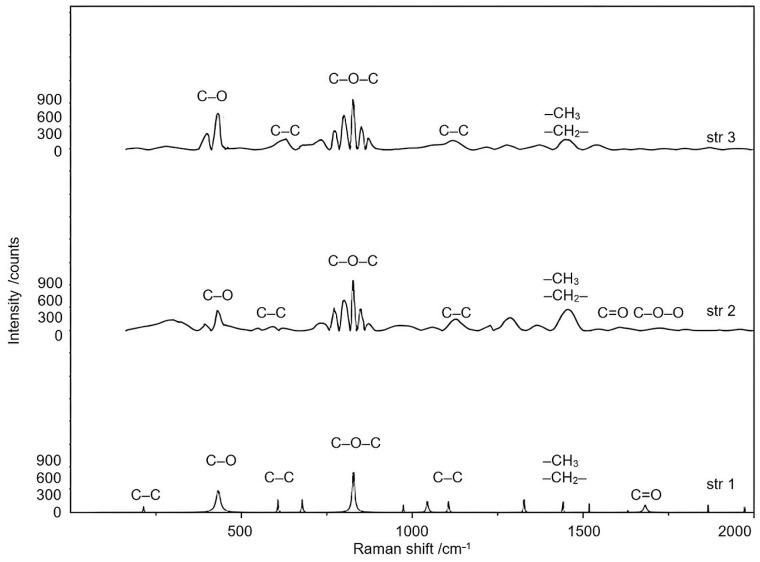
Raman spectra of samples.

Figure 7 reveals the Raman spectra of all three samples.

The Raman spectra of samples show weak bands at 215.1 cm^−^^1^ (str 1) and 279.4 cm^−^^1^ (str 3) that correspond to the cleavage of C-C aliphatic bonds, a band between 432.5 cm^−^^1^ (str 1) and 433.5 cm^−^^1^ (str 2 and str 3) characteristic for several types of alcohols [38], and bands between 596.2 cm^−^^1^ (str 2) and 635.7 cm^−^^1^ (str 3) attributed to the stretch of C-C alicyclic and aliphatic bonds. The C-O-C bond is indicated by the strong bands occurring between 827.4 and 829.9 cm^−^^1^. The bands at 1329.2 and 1442.6 cm^−^^1^ (str 1), 1449.9 cm^−^^1^ (str 2), and 1372.5 and 1460.6 cm^−^^1^ (str 3) are associated with the stretch and asymmetric cleavage of -CH_3_ and -CH_2_- bonds. The stretch of C=O bonds is indicated by the presence of moderate bands within the range of 1626.3 cm^−^^1^–1682.5 cm^−^^1^, while C-O-O- bonds were observed between 1757.8 cm^−^^1^ and 1770.8 cm^−^^1^.

J. Brzeska et al. [39] have synthesized and comparatively characterized different linear and branched aliphatic PUs. Like us, they also discovered that the -NH- signal is almost absent in Raman spectroscopy, and the spectra of all PU samples are quite similar.

### 3.7. Thermal Analysis

**Figure 8:**
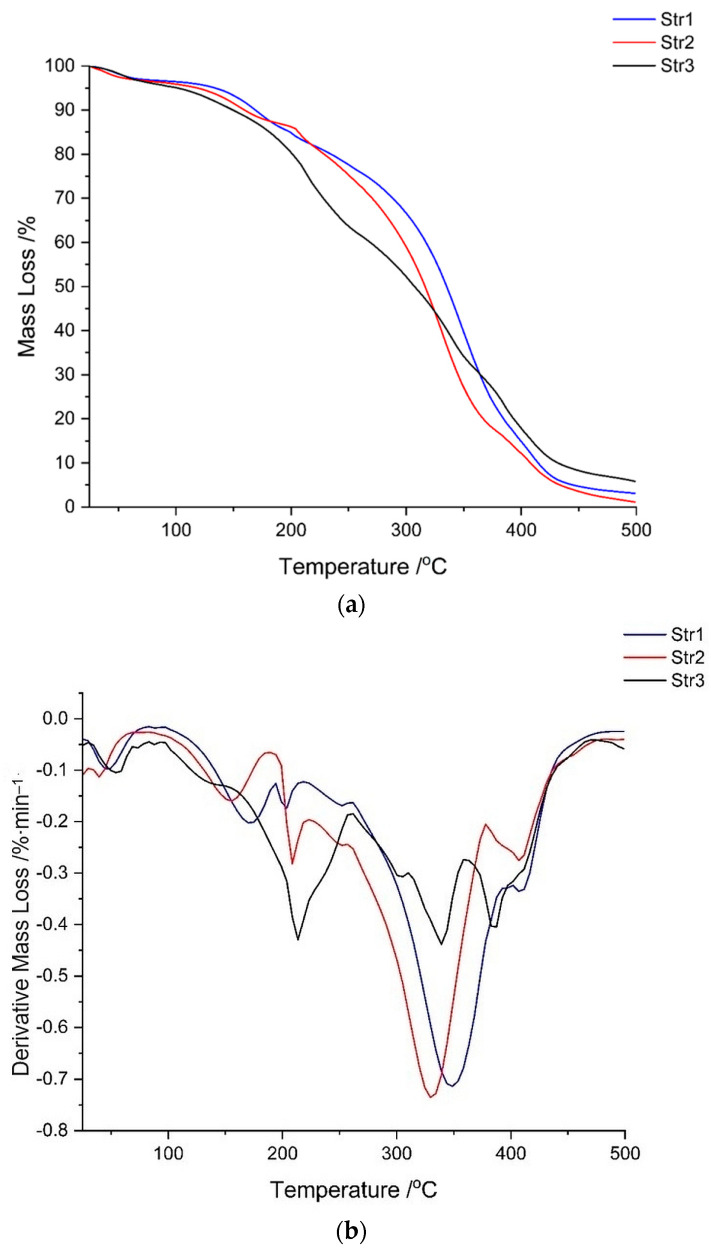
(**a**) TG and (**b**) DTG curves of samples.

**Figure 9:**
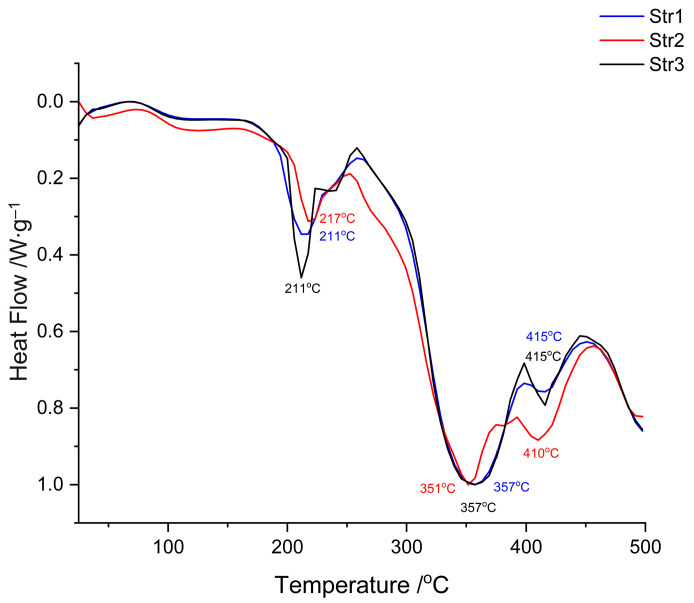
DSC curves of tested samples.

The following figures (Figure 8 and Figure 9) present the results of different thermal analyses—TG/DTG and DSC curves, respectively.

The thermal analysis (TG and DTG curves from Figure 8) shows that the decomposition of str 1 takes place within the same interval as for str 2 (an intense peak between 300 and 350 °C), while the third sample undergoes another two decomposition processes around 200 °C and 400 °C, respectively. A first stage of mass loss, below 100 °C for all samples, occurs due to water loss, while the decomposition between 200 and 400 °C can be attributed to the cleavage of PU linkage as previously described in the literature [40,41]. The decomposition of the carrier begins with a depolycondensation process and continues with the degradation of its hard segments (the part consisting of diisocyanate); the thermal degradation process is completed with the collapse of the soft segments from the macromolecular chains (the polyol part).

The DSC thermograms (Figure 9) confirm the data recorded in the previous thermal analysis. The samples are very similar, even if a different diisocyanate was used; two main endothermic peaks can be observed: a sharp one around 210 °C and a large one around 350 °C. The glass transition of these materials was not observed within this temperature range; the literature reports the Tg of polyurethanes to be at negative values, between −60 and −20 °C, depending on the hard/soft segments ratio [39,42].

### 3.8. Zetasizer

Dynamic light scattering techniques were employed to assess the size and polydispersity of particles (PDI), as well as their surface charge. Table 2 presents the values obtained in the Zetasizer evaluations.

The Zetasizer analyses (Table 2) have revealed the formation of dispersed systems containing two particle populations for samples str 1 and 3 and one population for str 2 (the percents indicate the size distribution by intensity); drug delivery systems based on dispersed samples often assure a prolonged release due to their different degradation rates [43]. The average size of particles was very similar, ranging between 132 and 190 nm; the PDI values also indicate a broad particles size distribution, which can have advantages and disadvantages: small nanocarriers with a narrow range of particle sizes are able to enter through pores of a capillary [44], while our particles with a large range of sizes present a balance between the amount of the active substance and the endocytosis-dependent cellular uptake based on various circulation times and further determining a decreased immunogenic response [45]. The values of the Zeta potential are specific to a colloidal system with moderate resistance against the tendency to form particle clusters; according to the literature, they are situated at the border between the values of nearly neutral and strongly cationic particles (28.3–31.5 mV) [46,47].

### 3.9. SEM Analysis

**Figure 10:**
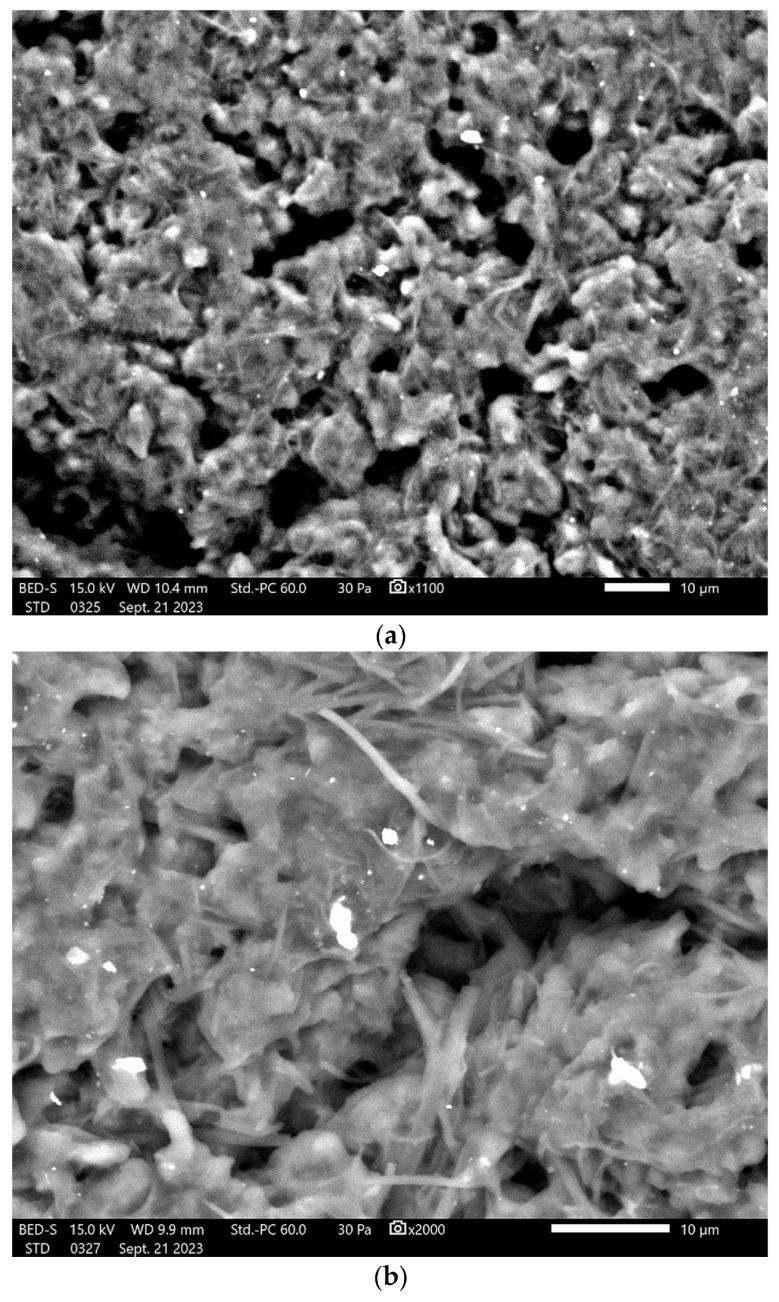

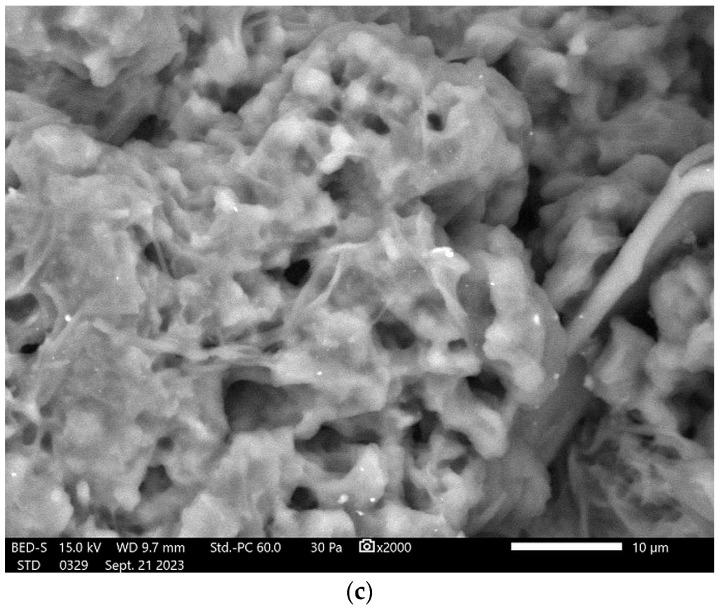
SEM images of tested samples: (**a**) str 1, (**b**) str 2, and (**c**) str 3.

Figure 10 shows the SEM images of the surface morphology and the shape of the particles of tested samples.

The adhesion of PU particles can be observed on dried samples (Figure 10), and this is a confirmation of the moderate resistance against the tendency to form clusters that was indicated by the values of the Zeta potential, but contrary to expectations, S. Samimi et al. [48] did not find a significant correlation between the Zeta potential and the stability of the particles. An option for decreasing this adhesion is to use larger amounts of the surfactant [22,23,24] or a mixture containing a lipophilic surfactant (Span^®^ 85) and a hydrophilic surfactant (Tween^®^ 20) [16].

### 3.10. Sample Stability

Generally, the suspensions of polymer drug delivery systems based on different nano- and microparticles are stable for months. The stability of samples is a key parameter that indicates the storage-controlled conditions such as humidity, temperature, sun exposure, etc. In the current study, the changes in sample color and pH were within a very narrow range for all three samples at the tested temperatures: the absorbance of samples changed between 0.72 and 1.14% (str 1), 0.53 and 0.90 (str 2), and 0.64 and 1.26% (str 3), while the pH was modified between 1.11 and 1.89% (str 1), 1.65 and 2.32% (str 2), and 1.57 and 2.20% (str 3). Table 3 displays the changes in the electrical conductivity; the values show the maximum percentages that were calculated as ratios between an instant measurement and the level from the first day.

### 3.11. Cell Viability

**Figure 11:**
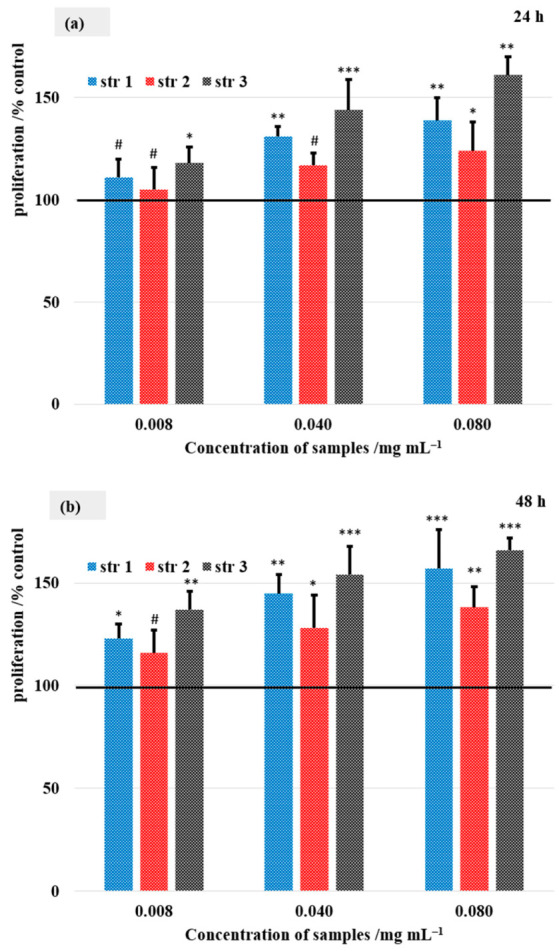

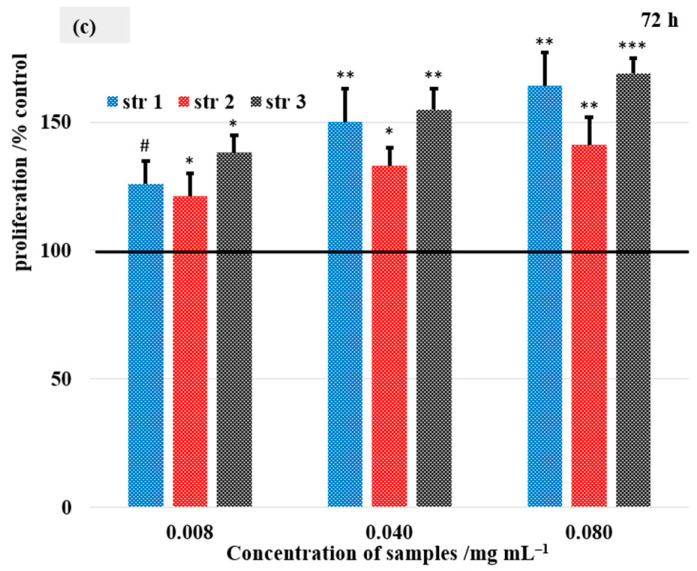
Cell proliferation rates after (**a**) 24, (**b**) 48, and (**c**) 72 h. Control was set to 100%, # *p* > 0.05, * *p* < 0.05, ** *p* < 0.01, *** *p* < 0.001. Bars represent mean ± standard error of the mean.

Figure 11 presents the HDFa cells’ proliferation rate.

The viability of the HDFa cells was determined at 24, 48, and 72 h after stimulation with the synthesized samples at a concentration of 0.008, 0.040, and 0.080 mg mL^−1^ to determine their cytotoxicity (Figure 11). After 24 h, a concentration-dependent increase in cell viability was found in all three samples. The highest values were observed at a concentration of 0.080 mg mL^−1^ (139% for str 1, 124% for str 2, and 161% for str 3).

At 48 h post-exposure, a similar trend was found in terms of influencing cell viability. Thus, a dose-dependent increase in cell viability was observed for all the tested compounds. Specifically, the maximum concentration tested for each compound increased cell viability to 157% (str 1), 138% (str 2), and 166% (str 3) vs. the control. At 72 h incubation, str 2 exhibited increased cell proliferation vs. the control (141%), however, it was to a smaller degree compared with the other two samples. In contrast, str 3 exhibited the highest values at the studied concentrations vs. control (138, 155, and 169%), while str 1 increased cell viability up to 164% vs. control.

### 3.12. Skin Irritation Evaluation

**Figure 12:**
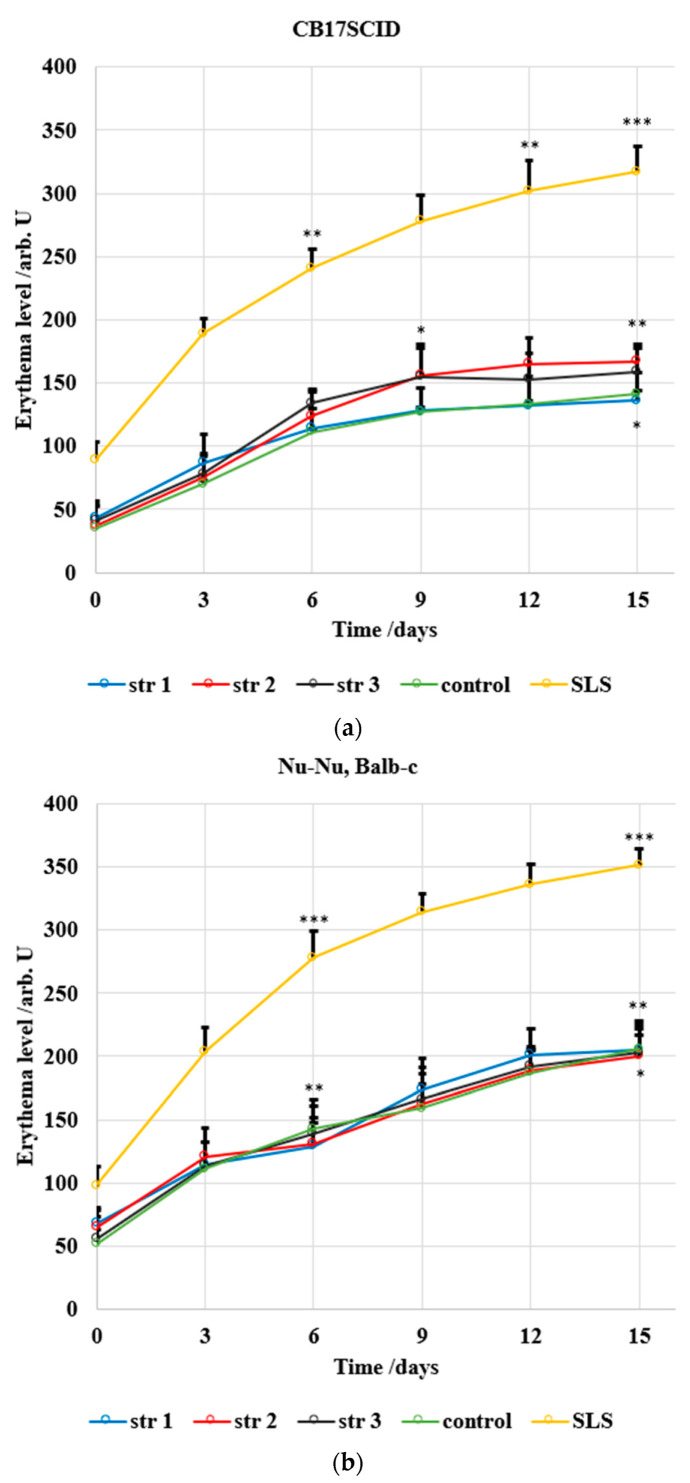
Evolution of erythema for (**a**) CB17SCID mice and (**b**) Balb-c, * *p* < 0.05, ** *p* < 0.01, *** *p* < 0.001. Bars represent mean ± standard error of the mean.

**Figure 13:**
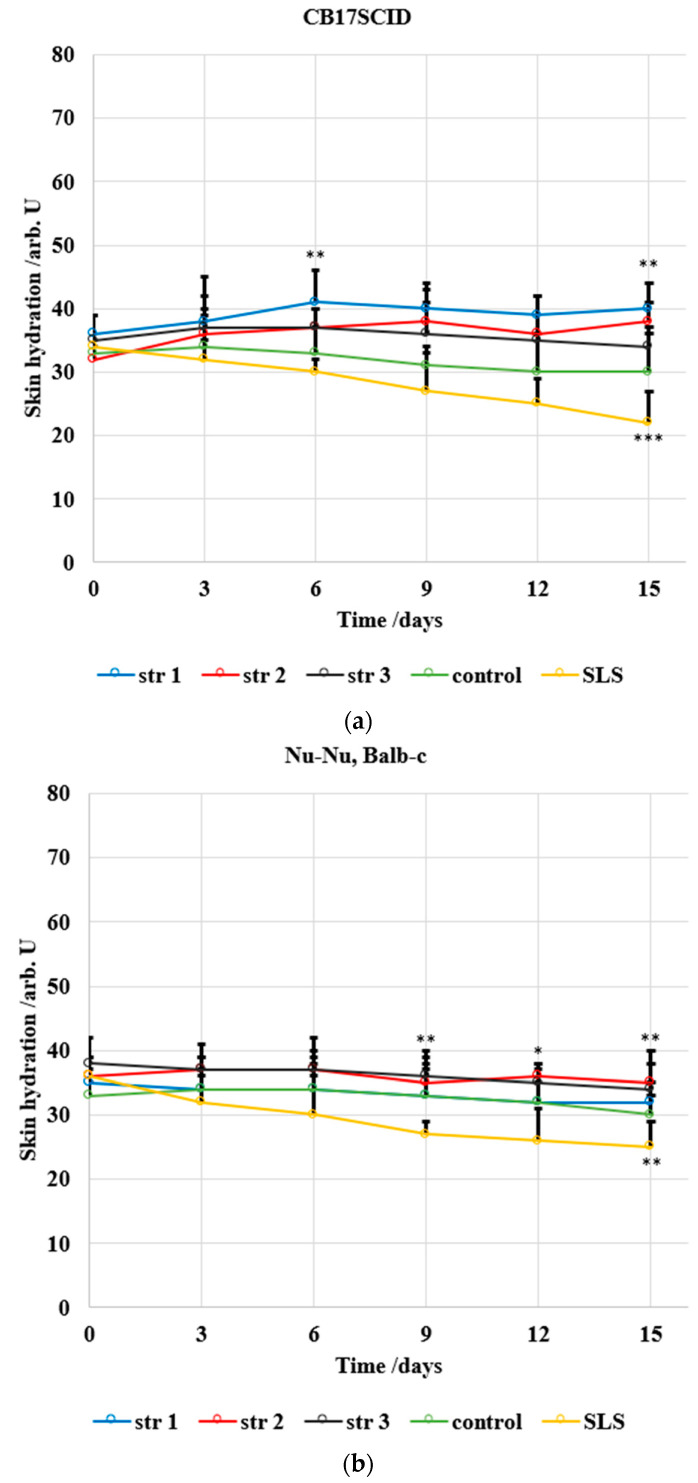
Evolution of the skin hydration for (**a**) CB17SCID mice and (**b**) Balb-c, * *p* < 0.05, ** *p* < 0.01, *** *p* < 0.001. Bars represent mean ± standard error of the mean.

Two different mice strains were used in this study due to their different skin sensitivity; Figure 12 and Figure 13 show the evolution of the main skin parameters (erythema and skin hydration) during the experiment.

Experimental mice represent successful research models in the development of new pharmaceuticals. The use of animals contributes to the advance of the scientific knowledge in developing and testing new drugs and therapies [49]. The mouse epidermis is often used as a model in skin pharmacology; skin irritations and cancer are studied on various strains of mice.

The following main parameters indicate a severe change in skin health: the increase in the transepidermal water loss and in the erythema level, combined with the decrease in skin hydration. Of note, these parameters may vary significantly within the time unit; not every change can be associated with a health impairment or a sign of illness—this is the reason why the tested compounds are comparatively assessed against a known irritative compound. The erythema assessment (Figure 12) shows an increased level for all samples and for both mice strains (CB17SCID and Nu-Nu, Balb-c); for the first strain, the increase was max. 130 arb. units/15 days for the PU samples—similar to the control group (106 arb. units/15 days), while sodium lauryl sulfate (which induces irritant contact dermatitis even at low concentrations, between 0.025% and 0.075%) has changed the erythema level by 228 arb. units/15 days. The differences from the 1st to the 15th day are much larger for Nu-Nu, Balb-c mice due to their increased sensitivity.

The level of skin hydration (Figure 13) has been modified only slightly in mice treated with the tested PU samples. The decrease in hydration was max. 1 arb. units/15 days in the case of CB17SCID mice compared with 12 arb. units/15 days (for mice treated with SLS) and max. 3 arb. units/15 days for Nu-Nu, Balb-c mice compared with 10 arb. units/15 days (for mice treated with SLS).

## 4. Discussion

Polymers are very frequently used as carrier materials in the administration of many drugs that pose various challenges such as low solubility and/or bioavailability, fast degradation, etc. [50,51]. The beginning of PU application in the pharmaceutical research field can be dated to the last two decades of the last century, when a group from Japan studied and reported on the release behavior of crystal violet from a polyurethane gel [52], as well as the release of an antituberculous mixture (isoniazid, rifampicin, novocain) from a PU foam used as drug carrier matrixes [53].

In the current study, three aliphatic diisocyanates with different chemical structures were used to develop a PU drug delivery system for dCMP. The samples have been characterized by physico-chemical, in vitro, and in vivo evaluations in order to establish the influence of the structure of the precursor on the properties of the final formulation. Sample str 1 contains a straight-chain diisocyanate, str 2 is based on a cyclic isocyanate, while the third sample was synthesized using a diisocyanate-ester (LDI is found as ethyl-2,6-diisocyanatohexanoate).

The pH of the synthesized samples, between 6.6 and 6.8 and measured in aqueous solutions (0.8 mg mL^−1^), is specific to nearly neutral products aimed at various administration routes. Generally, the pH of new formulations is maintained between 6 and 8 by using various buffer solutions in order to avoid altering the normal functioning of systems and tissues [54]. The narrow range of the refractivity index (1.59–1.61) for the three samples indicates their similarity; the literature is rather sparse in terms of values of the refractivity index for polymer drug carriers, but M. Iwazumi and A. Schneider reported a larger range (1.53–1.63) for PU coatings [55], while J. Bauer et al. have reported higher values (1.55–1.75) for PU elastomer samples based on methylene-diphenyl diisocyanate and hexamethylene diisocyanate, respectively [56].

The aqueous solubility represents a significant drawback for PU carriers; we previously revealed this challenge [57], which was since improved only to a small extent. Thus, the water solubility of samples ranges between 0.84 and 0.90 mg mL^−1^ in acetone (1.04–1.11 mg mL^−1^) and in DMSO (1.02–1.06 mg mL^−1^). Generally, drug carriers that show low aqueous solubilities are associated with delayed release [58], but this parameter is also influenced by the chemical structure of the delivery system according to the literature [59].

High values were found in the tested samples for the of dCMP encapsulation efficacy (between 67 and 69%); this parameter plays a major role in the design of drug delivery systems, and it depends on the microencapsulation technique, the solubility of raw materials, the particle size, and the yield of the synthesis reaction. Another investigation on a PU carrier has revealed values of the encapsulation efficacy in a broad range between 55 and 85% [60], while Y. Batyrbekov and R. Iskakov [61] reported an even wider range for the values of entrapment efficiency of PU delivery systems (40–91%). The different structure of the isocyanates that were used as raw materials seems to not have an influence on the encapsulation efficacy, and this can be explained by the similarity of the particle sizes on the one hand and by the same synthesis procedure on the other hand, with both parameters being mentioned in the literature [62].

The cumulative drug release shows a similar trend that indicates the similarity between samples despite the particular isocyanate used. Almost 15% of the total amount was released after 48 h and around 45% after 5 days, in which the samples were maintained in a degrading environment (a simulated body fluid previously described by T. Kokubo [26]). The kinetics can be described as a near-linear release specific to a non-Fickian diffusion that was already met in other PU materials [63]. N. Abbasnezhad et al. have also reported a non-Fickian diffusion for PU films loaded with diclofenac, but the mechanism depends on the film thickness [64].

The investigation of artificial membrane permeability is based on an artificial membrane, such as polyvinylidene difluoride, which is widely used for immunoblotting techniques, and two compartments of the so-called acceptor and donor chambers. The measurements provide different values for the permeability coefficients, known as percentage of flux or transported solute, which represents the part of the tested sample in the acceptor chamber. It was found that the particles’ penetrability through the artificial membrane was up to 50% in the first ten hours. It is currently known that the impermeability of the biological membranes is a major disadvantage in drug delivery due to its influence on the bioavailability of many drugs; the diffusion rate depends on particles’ solubility, their size, and their surface charge. In the current study, almost the entire amount passed through the artificial membrane within 60 h.

The FT-IR evaluation was performed to find the possible interactions between the encapsulated substance and the polyurethane drug delivery system, as well as to gain more data about the influence of the aliphatic diisocyanate structure on the properties of every sample; no significant difference was observed between the samples. Additionally, there are no traces of -NCO peaks (2270 cm^−1^) detected in the three samples obtained via a polyaddition process, and this indicates that the entire amount of diisocyanate has reacted completely to form polyurethane; the toxicity of isocyanate functional groups is widely known after the Bhopal gas tragedy in December 1984, when more than 500,000 people were exposed to methyl isocyanate [65].

Raman spectroscopy was also used to assess the chemical structures of products and possible interactions. The complete reaction of isocyanate groups can be assumed by the presence of the -C-N- single bond axial stretch (between 1518–1550 cm^−1^), because the specific peak of -NCO groups does not appear in the observed range [66].

The investigation of the thermal behavior of polyurethane samples is very important due to the potentially hazardous chemicals that are released: a yellow smoke containing hydrogen cyanide and other toxic products was reported at temperatures above 600 °C [67]. The composition of the volatile compounds and residues arising from PU thermal decomposition is influenced by the conditions of synthesis and the nature of the raw materials [68]. However, the safety of PU biomaterials is not a concern due to their high stability up to 300 °C according to the literature [69]. By inspecting the thermogravimetric curves of the current samples, a significant thermal stability was observed up to 200 °C; the investigation of the thermal decomposition above 200 °C is not relevant for materials intended for used in biomedical related applications, but it is worthwhile because of the study of macromolecular chains (hard vs. soft segments) and to predict the half-life of a pharmaceutical formulation [70] or the compatibility between a drug and various excipients [71].

An assessment based on particles’ velocity was included in order to find the particles’ distribution size with regard to the electrophoretic mobility to analyze their tendency to form agglomerations. Repeated measurements on every sample have revealed that an isocyanate structure does not affect the observed parameters significantly. The dimensions of PU particles are between the limits recommended by the literature [72], and the values of the polydispersity index are highly corelated with the Zeta potentials. Most studies on the surface of particles have shown that the charge influences the ability to pass through membranes. The Zeta potential values of PU-based materials are found in a large range (between −20 and +30 mV) according to Y. Ren et al. [73]; the positive values observed in the current samples can be related to the absence of free hydroxyl and carboxyl groups and to the presence of THAM as a crosslinker.

In order to characterize the surface of PU particles, SEM analysis was employed; Figure 10 shows semi-crystalline samples based on a mixture of individual particles and agglomerates, which confirm the various populations found in the Zetasizer analysis.

The stability of pharmaceutical formulations is a very important parameter for the preservation of the active drug’s bioavailability and to avoid chemical changes that may increase its toxic potential. Polyurethanes are very stable materials—a natural degradation of foams becomes visible only after 20–30 years [74]. However, there are known bacterial, thermal-, and photo-oxidative processes that might contribute to their faster degradation. The relation between the samples’ stability and their electrical conductivity was described in the literature a long time ago [75]: the sedimentation and the streaming potential, electrophoresis, and electroosmosis represent a few of the properties of colloidal suspensions that can be changed over the time. On the other hand, the Gibbs–Donnan effect, related to the diffusion of charged particles through semi-permeable membranes, is also influenced by the electrical conductivity of the sample. The current results indicate that the employed carrier is stable between 8 and 40 °C at 65% humidity for 30 days, which represents a similar behavior to other PU samples described in the literature [23].

Dermal fibroblasts are specialized cells, located deep in the skin, which generate connective tissue and help it to recover after an injury. The fibroblasts are often used in the evaluation of collagen production, identification of RNA mutations, and cytotoxicity as an indicator of bio-incompatibility [76]. Following 24 h, 48 h, and 72 h stimulation with the tested compounds, the cell viability increased in a time- and dose-dependent manner. The highest values of cell proliferation were obtained in the case of the str 3 sample tested at the highest concentration. To the best of our knowledge, no studies on the effect of PU-based carriers for nucleosides on HDFa cells have been performed. However, PUs have been shown to stimulate cells’ adhesion and proliferation at certain concentrations. In a study by Zanetta and colleagues, it was found that PU foams used as scaffolds can increase the viability of MG-63 and mesenchymal stem cells [77]. González-García et al. [78] described the cytocompatibility of two PU-based biomaterials used for bone tissue regeneration. Their results from a 9-day assessment on the human osteoblastic cells indicated excellent cell adhesion to the surface of a material that was fully covered by cells and a good proliferation of cells. Additionally, in a previous study by our team, the effect of a PU carrier loaded with ursolic and oleanolic acids on skin tumors was investigated, and the results showed that for the sample with encapsulated acids, the cells’ viability was slightly higher than in the sample with free acids [79]. Based on the obtained results, it can be stated that this PU delivery system could be considered a good possible choice for further in vivo evaluation of carriers used for nucleosides transfer.

An in vivo non-invasive and quantitative study of erythema and skin hydration level was included in this study to investigate if the synthesized products present irritative effects. M. Denzinger et al. consider that modifications in the level of skin hydration are detectable before the appearance of erythema changes [80]. Despite the slight increase in erythema and decrease in skin hydration during assessment, the results obtained in this study indicate that the synthesized products do not alter the epidermal function with regard to the strong correlation between two skin parameters. A. Chioreanu et al. describe a similar drug delivery system based on polyester-urethane microparticles used for curcumin, and they report similar differences in skin parameters between the samples and reference (sodium lauryl sulfate) [81].

Collectively, the physico-chemical, in vitro, and in vivo assessments of the currently synthesized drug delivery systems have revealed that these products are very similar, their properties are not influenced by the isocyanate structure, and they are safe to use in further clinical trials. Of course, it is not possible to clearly elucidate all beneficial and harmful effects, despite the promising results of this pioneering study. The main limitations of the current research are the insufficient control of the release kinetics and a low level of sample solubility.

The polyurethane-based drug delivery systems have multiple benefits: the particles may assure a controlled release based on the polyester/polyether ratio, and their size can be easily adjusted via the chain extender amount. However, the current study presents a few limitations. Unfortunately, the potential improvement in the potency and therapeutic efficacy is difficult to estimate due to the lack of research on the activity of pure dCMP. Broader particle size distributions and PDI values bigger than 1.0 were also found in other studies [82,83] on polyurethane biomaterials based on polycaprolactone, but dynamic light scattering is a technique that often leads to discrepancies in particles size, typically larger than 10%, when the dispersity of a sample is high. Surface charge is one of the important properties of particles that can determine the adhesion onto surfaces; therefore, a comparative evaluation of charged particles and charge-neutral particles is necessary. Furthermore, the characterization of the products must be continued for a better understanding of the intermolecular interactions between the active substance and the carrier.

## 5. Conclusions

Polyurethanes are often found in many industries under various forms, from elastomers and rigid foams, coatings and insulation materials, to heart valves, artificial skin, arteries and veins, wound dressing materials, and long-term implants. This paper introduces the synthesis and preliminary characterization of a PU drug delivery system that combines different degradation pathways (hydrolytic and enzymatic types based on the ester functional groups, oxidative, physical and enzymatic types based on the ether groups, and enzymatic and hydrolytic types based on the urethane functional groups) in order to obtain a drug carrier with a controlled release. The characterization of the samples indicates the preparation of a carrier able to encapsulate large amounts of nucleosides, with a prolonged release and good penetrability rate.

The inexpensive materials and the mild reaction conditions that are specific to the sustainable or green chemistry on the one hand, combined with the easiness of modulating the particle size by changing the ratio between the main polyol/polyols and the chain extenders on the other hand, make these polymers attractive materials for drug delivery. Their biodegradability and long-term biocompatibility, already validated by previous studies, add to their potential use as drug carriers.

Overall, all the intended outcomes of the current research, related to obtaining a PU carrier for dCMP and to the comparative assessment of samples based on different isocyanate structures, have been achieved. The future perspectives of this study for practical applications consist of a deep analysis on the modification of the macromolecular chains in order to increase the carrier solubility and on the toxicity of the degradation products.

## Figures and Tables

**Table 1:** The solubility of samples.

Sample	Solubility (mg mL^−1^)		
	Water	Acetone	DMSO
str 1	0.86 ± 0.08	1.04 ± 0.05	1.05 ± 0.06
str 2	0.84 ± 0.05	1.11 ± 0.04	1.02 ± 0.10
str 3	0.90 ± 0.06	1.09 ± 0.09	1.06 ± 0.08

*p* < 0.05 (str 1 vs. str 2), *p* = 0.141 (str. 1 vs. str. 3), and *p* < 0.05 (str. 2 vs. str 3).

**Table 2:** The Zetasizer characterization of samples.

Sample	Analyzed Parameters		
	Size (nm)	PDI	Zeta Potential (mV)
str 1	186 ± 11 (81%) 145 ± 7 (19%)	1.04 ± 0.02	31.5 ± 1.7
str 2	164 ± 14 (100%)	0.94 ± 0.01	28.3 ± 1.2
str 3	190 ± 12 (67%) 132 ± 17 (33%)	1.09 ± 0.02	30.0 ± 1.6

**Table 3:** The changes in the electrical conductivity after 30 days.

Sample	Percentual Changes at Different Temperatures, %		
	8 ± 0.5 °C	25 ± 0.5 °C	40 ± 0.5 °C
str 1	7.65 ± 0.18	7.08 ± 0.09	6.32 ± 0.14
str 2	5.32 ± 0.14	6.59 ± 0.11	6.11 ± 0.21
str 3	6.16 ± 0.12	5.91 ± 0.19	7.45 ± 0.20

*p* < 0.05 (str 1 vs. str 2), *p* = 0.058 (str. 1 vs. str. 3), and *p* = 0.081 (str. 2 vs. str 3).

## Data Availability

No new data were created or analyzed in this study. Data sharing is not applicable to this article.

## References

[1] Huang R.M., Chen Y.N., Zeng Z., Gao C.H., Su X., Peng Y. (2014). Marine nucleosides: Structure, bioactivity, synthesis and biosynthesis. Mar. Drugs.

[2] Fu J., Wu Z., Zhang L., Teplow D.B. (2019). Clinical applications of the naturally occurring or synthetic glycosylated low molecular weight drugs. Progress in Molecular Biology and Translational Science.

[3] Yegutkin G.G. (2021). Adenosine metabolism in the vascular system. Biochem. Pharmacol..

[4] Villa E., Ali E.S., Sahu U., Ben-Sahra I. (2019). Cancer cells tune the signaling pathways to empower de novo synthesis of nucleotides. Cancers.

[5] Rawat R.S., Kumar S. (2023). Understanding the mode of inhibition and molecular interaction of taxifolin with human adenosine deaminase. J. Biomol. Struct. Dyn..

[6] Teng H., Wang Y., Sui X., Fan J., Li S., Lei X., Shi C., Sun W., Song M., Wang H. (2023). Gut microbiota-mediated nucleotide synthesis attenuates the response to neoadjuvant chemoradiotherapy in rectal cancer. Cancer Cell.

[7] Tan C.L. (2022). Facts Cannot be Ignored When Considering the Origin of Life# 2: Challenges in Generating the First Gene-encoding Template DNA or RNA. Answ. Res. J..

[8] Martikainen L., Walther A., Ikkala O. Cytidine Functionalization Promotes Synergistic Mechanical Properties in Nacre-Mimetic Nanocomposites. Proceedings of the International Conference on Composite Materials.

[9] Studzińska S., Buszewski B. (2013). Effect of mobile phase pH on the retention of nucleotides on different stationary phases for high-performance liquid chromatography. Anal. Bioanal. Chem..

[10] Momparler R.L., Rossi M., Bouchard J., Vaccaro C., Momparler L.F., Bartolucci S. (1984). Kinetic interaction of 5-AZA-2’-deoxycytidine-5’-monophosphate and its 5’-triphosphate with deoxycytidylate deaminase. Mol. Pharmacol..

[11] Zlatev I., Giraut A., Morvan F., Herdewijn P., Vasseur J.J. (2009). δ-Di-carboxybutyl phosphoramidate of 2′-deoxycytidine-5′-monophosphate as substrate for DNA polymeri-zation by HIV-1 reverse transcriptase. Bioorg. Med. Chem..

[12] Rusu L.-C., Ardelean L.C., Jitariu A.-A., Miu C.A., Streian C.G. (2020). An Insight into the Structural Diversity and Clinical Applicability of Polyurethanes in Biomedicine. Polymers.

[13] Wendels S., Avérous L. (2021). Biobased polyurethanes for biomedical applications. Bioact. Mater..

[14] Gawlikowski M., El Fray M., Janiczak K., Zawidlak-Węgrzyńska B., Kustosz R. (2020). In-vitro biocompatibility and hemocompatibility study of new PET copolyesters intended for heart assist devices. Polymers.

[15] Singh S., Kumar Paswan K., Kumar A., Gupta V., Sonker M., Ashhar Khan M., Kumar A., Shreyash N. (2023). Recent Advancements in Polyurethane-based Tissue Engineering. ACS Appl. Bio Mater..

[16] Bouchemal K., Briançon S., Perrier E., Fessi H., Bonnet I., Zydowicz N. (2004). Synthesis and characterization of polyurethane and poly(ether urethane) nanocapsules using a new technique of interfacial polycondensation combined to spontaneous emulsification. Int. J. Pharm..

[17] Bouchemal K., Briançon S., Perrier E., Fessi H. (2004). Nano-emulsion formulation using spontaneous emulsification: Solvent, oil and surfactant optimisation. Int. J. Pharm..

[18] Bouchemal K., Briançon S., Couenne F., Fessi H., Tayakout M. (2006). Stability studies on colloidal suspensions of polyurethane nanocapsules. J. Nanosci. Nanotechnol..

[19] Niu Y., Zhang B., Galluzzi M. (2021). An amphiphilic aggregate-induced emission polyurethane probe for in situ actin observation in living cells. J. Colloid Interface Sci..

[20] Seley-Radtke K.L., Yates M.K. (2018). The evolution of nucleoside analogue antivirals: A review for chemists and non-chemists. Part 1: Early structural modifications to the nucleoside scaffold. Antivir. Res..

[21] LiverTox: Clinical and Research Information on Drug-Induced Liver Injury. https://www.ncbi.nlm.nih.gov/books/NBK548938/.

[22] Borcan L.-C., Dudas Z., Len A., Fuzi J., Borcan F., Tomescu M.C. (2018). Synthesis and characterization of a polyurethane carrier used for a prolonged transmembrane transfer of a chili pepper extract. Int. J. Nanomed..

[23] Borcan F., Chirita-Emandi A., Andreescu N.I., Borcan L.-C., Albulescu R.C., Puiu M., Tomescu M.C. (2019). Synthesis and preliminary characterization of polyurethane nanoparticles with ginger extract as a possible cardiovascular protector. Int. J. Nanomed..

[24] Munteanu M.F., Ardelean A., Borcan F., Trifunschi S.I., Gligor R., Ardelean S.A., Coricovac D., Pinzaru I., Andrica F., Borcan L.-C. (2017). Mistletoe and Garlic Extracts as Polyurethane Carriers—A Possible Remedy for Choroidal Melanoma. Curr. Drug Deliv..

[25] Borcan F., Len A., Bordejevic D.A., Dudas Z., Tomescu M.C., Valeanu A.N. (2020). Obtaining and characterization of a polydisperse system used as a transmembrane carrier for isosorbide derivatives. Front. Chem..

[26] Borcan F., Len A., Dehelean C.A., Dudás Z., Ghiulai R., Iftode A., Racoviceanu R., Soica C.M. (2021). Design and Assessment of a Polyurethane Carrier Used for the Transmembrane Transfer of Acyclovir. Nanomaterials.

[27] Tuta-Sas I., Borcan F., Sas I. (2023). Synthesis and preliminary characterization of polyurethane matrices used as a drug carrier for bromelain. Mater. Plast..

[28] Citu C., Ceuta L., Popovici R., Ionescu D., Pinzaru I., Borcan F. (2015). Alternative Possibilities to Asses a Phytohormone Release Rate from a Polyurethane Carrier. Mater. Plast..

[29] Moleriu L., Duse A.O., Borcan F., Soica C., Kurunczi L., Nicolov M., Mioc M. (2017). Formulation and characterization of antibacterial hydrogels based on polyurethane microstructures and 1,2,4-triazole derivatives. Mater. Plast..

[30] Garms B.C., Poli H., Baggley D., Han F.Y., Whittaker A.K., Anitha A., Grondahl L. (2021). Evaluating the effect of synthesis, isolation, and characterisation variables on reported particle size and dispersity of drug loaded PLGA nanoparticles. Mater. Adv..

[31] Baino F., Yamaguchi S. (2020). The Use of Simulated Body Fluid (SBF) for Assessing Materials Bioactivity in the Context of Tissue Engineering: Review and Challenges. Biomimetics.

[32] Kumar P., Ganure A.L., Subudhi B.B., Shukla S. (2015). Design and Comparative Evaluation of In-vitro Drug Release, Pharmacokinetics and Gamma Scintigraphic Analysis of Controlled Release Tablets Using Novel pH Sensitive Starch and Modified Starch- acrylate Graft Copolymer Matrices. Iran. J. Pharm. Res..

[33] Kamiloglu S., Sari G., Ozdal T., Capanoglu E. (2020). Guidelines for cell viability assays. Food Front..

[34] Vázquez-Blanco S., González-Freire L., Dávila-Pousa M.C., Crespo-Diz C. (2018). pH determination as a quality standard for the elaboration of oral liquid compounding formula. Farm. Hosp..

[35] Zeng L., An L., Wu X. (2011). Modeling drug-carrier interaction in the drug release from nanocarriers. J. Drug Deliv..

[36] Baysal G., Aydın H., Hoşgören H., Uzan S., Karaer H. (2016). Improvement of Synthesis and Dielectric Properties of Polyurethane/Mt-QASs+ (Novel Synthesis). J. Polym. Environ..

[37] Sit I., Young M.A., Kubicki J.D., Grassian V.H. (2023). Distinguishing different surface interactions for nucleotides adsorbed onto hematite and goethite particle surfaces through ATR-FTIR spectroscopy and DFT calculations. Phys. Chem. Chem. Phys..

[38] Lin-Vien D., Colthup N.B., Fateley W.G., Grasselli J.G., Lin-Vien D., Colthup N.B., Fateley W.G., Grasselli J.G. (1991). Alcohols and Phenols. The Handbook of Infrared and Raman Characteristic Frequencies of Organic Molecules.

[39] Brzeska J., Elert A.M., Morawska M., Sikorska W., Kowalczuk M., Rutkowska M. (2018). Branched Polyurethanes Based on Synthetic Polyhydroxybutyrate with Tunable Structure and Properties. Polymers.

[40] Lopes R.V.V., Osorio L.F.B., Santos M.L., Sales M.J.A. (2012). Characterization of Polyurethanes from Vegetable Oils by TG/DTG, DMA and FT-IR. Macromolec. Symp..

[41] Oprea S., Vlad S. (2007). Polyurethane Materials Using Aliphatic Diisocyanates for Passive Isolation in Buildings Applications. Mater. Plast..

[42] Son T.W., Lee D.W., Lim S.K. (1999). Thermal and Phase Behavior of Polyurethane Based on Chain Extender, 2,2-Bis-[4-(2-hydroxyethoxy )phenyl]propane. Polym. J..

[43] Peterson G.I., Ko W., Hwang Y.J., Choi T.L. (2020). Mechanochemical Degradation of Amorphous Polymers with Ball-Mill Grinding: Influence of the Glass Transition Temperature. Macromolecules.

[44] Townsley M.I., Parker J.C., Longenecker G.L., Perry M.L., Pitt R.M., Taylor A.E. (1988). Pulmonary embolism: Analysis of endothelial pore sizes in canine lung. Am. J. Physiol. Heart Circ. Physiol..

[45] Danaei M., Dehghankhold M., Ataei S., Hasanzadeh Davarani F., Javanmard R., Dokhani A., Khorasani S., Mozafari M.R. (2018). Impact of particle size and polydispersity index on the clinical applications of lipidic nanocarrier systems. Pharmaceutics.

[46] Salopek B., Krasic D., Filipovic S. (1992). Measurement and application of zeta-potential. Rud. Geol. Naft. Zb..

[47] Clogston J.D., Patri A.K. (2011). Zeta potential measurement. Methods Mol. Biol..

[48] Samimi S., Maghsoudnia N., Baradaran Eftekhari R., Dorkoosh F., Mohapatra S.S., Ranjan S., Dasgupta N., Mishra R.K., Thomas S. (2019). Chapter 3—Lipid-Based Nanoparticles for Drug Delivery Systems. Micro and Nano Technologies, Characterization and Biology of Nanomaterials for Drug Delivery.

[49] Lemon R., Dunnett S.B. (2005). Surveying the literature from animal experiments. BMJ.

[50] Begines B., Ortiz T., Pérez-Aranda M., Martínez G., Merinero M., Argüelles-Arias F., Alcudia A. (2020). Polymeric Nanoparticles for Drug Delivery: Recent Developments and Future Prospects. Nanomaterials.

[51] Elmowafy M., Shalaby K., Elkomy M.H., Alsaidan O.A., Gomaa H.A.M., Abdelgawad M.A., Mostafa E.M. (2023). Polymeric Nanoparticles for Delivery of Natural Bioactive Agents: Recent Advances and Challenges. Polymers.

[52] Kohjiya S., Ikeda Y., Takesako S., Yamashita S. (1991). Drug release behavior from polyurethane gel. React. Polym..

[53] Batyrbekov E.O., Moshkevich S.A., Rukhina L.B., Bogin R.A., Zhubanov B.A. (1990). Some fields of biomedical application of polyurethanes. Polym. Int..

[54] Abdella S., Abid F., Youssef S.H., Kim S., Afinjuomo F., Malinga C., Song Y., Garg S. (2023). pH and its applications in targeted drug delivery. Drug Discov. Today.

[55] Iwazumi M., Schneider A. (2011). High Refractive Index Aqueous Polyurethane Dispersion Coating Compositions.

[56] Bauer J., Gutke M., Heinrich F., Edling M., Stoycheva V., Kaltenbach A., Burkhardt M., Gruenefeld M., Gamp M., Gerhard C. (2020). Novel UV-transparent 2-component polyurethane resin for chip-on-board LED micro lenses. Opt. Mater. Express.

[57] Citu I.M., Borcan F., Zambori C., Tita B., Paunescu V., Ardelean S. (2015). Influence of crosslinking agent—Chain extender ratio on the properties of hyperbranched polyurethane structures used as dendritic drug carrier. Rev. Chim. Buchar..

[58] Tekade A.R., Yadav J.N. (2020). A Review on Solid Dispersion and Carriers Used Therein for Solubility Enhancement of Poorly Water Soluble Drugs. Adv. Pharm. Bull..

[59] Józó M., Simon N., Yi L., Móczó J., Pukánszky B. (2022). Improved Release of a Drug with Poor Water Solubility by Using Electrospun Water-Soluble Polymers as Carriers. Pharmaceutics.

[60] Javidi M., Fathi Fathabadi H., Jenabali Jahromi S.A., Khorram M. (2019). Investigating the interfacial synthesis of polyurethane microcapsules and optimization of the process using response surface method. Mater. Res. Express.

[61] Batyrbekov Y., Iskakov R., Zafar F., Sharmin E. (2012). Polyurethane as Carriers of Antituberculosis Drugs. Polyurethane.

[62] Othman N., Masarudin M.J., Kuen C.Y., Dasuan N.A., Abdullah L.C., Md. Jamil S.N.A. (2018). Synthesis and Optimization of Chitosan Nanoparticles Loaded with l-Ascorbic Acid and Thymoquinone. Nanomaterials.

[63] Fu Y., Kao W.J. (2010). Drug release kinetics and transport mechanisms of non-degradable and degradable polymeric delivery systems. Expert Opin. Drug. Deliv..

[64] Abbasnezhad N., Zirak N., Shirinbayan M., Kouidri S., Salahinejad E., Tcharkhtchi A., Bakir F. (2020). Controlled release from polyurethane films: Drug release mechanisms. J. Appl. Polym. Sci..

[65] Varma R., Varma D.R. (2005). The Bhopal Disaster of 1984. Bull. Sci. Technol. Soc..

[66] Da Cunha F.O.V., Melo D.H.R., Veronese V.B., Forte M.M.C. (2004). Study of castor oil polyurethane-poly(methyl methacrylate) semi-interpenetrating polymer network (SIPN) reaction parameters using a 2^3^ factorial experimental design. Mater. Res. Ibero-Am..

[67] Chambers J., Jirickny J., Reese C. (1981). The thermal decomposition of polyurethanes and polyisocyanurates. Fire Mater..

[68] Amado J.C.Q., Evingür G.A., Pekcan O., Achilias D.S. (2023). Thermal Resistance Properties of Polyurethanes and Its Composites. Thermosoftening Plastics.

[69] Bolcu C., Borcan F., Nutiu R. (2006). Aspects regarding the synthesis and characterization of some types of thermoplastic polyurethanes. Mater. Plast..

[70] Tita B., Fulias A., Marian E., Tita D. (2009). Thermal Stability and Decomposition Kinetics Under Non-isothermal Conditions of Sodium Diclofenac. Rev. Chim..

[71] Omari D., Sallam A., Al-Hmoud H., Rashid I. (2023). Modafinil-excipient compatibility study using differential scanning calorimetry. J. Adv. Pharm. Technol. Res..

[72] Peng T., Lin S., Niu B., Wang X., Huang Y., Zhang X., Li G., Pan X., Wu C. (2016). Influence of physical properties of carrier on the performance of dry powder inhalers. Acta Pharm. Sin. B.

[73] Ren Y., Zhou H., Lu J., Huang S., Zhu H., Li L. (2020). Theoretical and Experimental Optimization of the Graft Density of Functionalized Anti-Biofouling Surfaces by Cationic Brushes. Membranes.

[74] Bhuvaneswari H., Sabu T., Ajay V.R., Krishnan K., Abitha V.K., Martin G.T. (2018). Degradability of Polymers. Recycling of Polyurethane Foams.

[75] Woxholt W.G. (1959). Electrical Conductivity and the Stability of Colloids. Am. Water Work. Assoc..

[76] Schmidt R.J., Chung L.Y., Andrews A.M., Turner T.D. (1993). Toxicity of L-ascorbic acid to L929 fi-broblast cultures: Relevance to biocompatibility testing of materials for use in wound management. J. Biomed. Mater. Res..

[77] Zanetta M., Quirici N., Demarosi F., Tanzi M.C., Rimondini L., Farè S. (2009). Ability of polyurethane foams to support cell prolif-eration and the differentiation of MSCs into osteoblasts. Acta Biomater..

[78] González-García D.M., Marcos-Fernández Á., Rodríguez-Lorenzo L.M., Jiménez-Gallegos R., Vargas-Becerril N., Téllez-Jurado L. (2018). Synthesis and in Vitro Cytocompatibility of Segmented Poly(Ester-Urethane)s and Poly(Ester-Urea-Urethane)s for Bone Tissue Engineering. Polymers.

[79] Oprean C., Borcan F., Pavel I., Dema A., Danciu C., Soica C., Dehelean C., Nicu A., Ardelean A., Cristea M. (2016). In Vivo Biological Evaluation of Polyurethane Nanostructures with Ursolic and Oleanolic Acids on Chemically-induced Skin Carcinogenesis. In Vivo.

[80] Denzinger M., Krauss S., Held M., Joss L., Kolbenschlag J., Daigeler A., Rothenberger J. (2020). A quantitative study of hydration level of the skin surface and erythema on conventional and microclimate management capable mattresses and hospital beds. J. Tissue Viability.

[81] Chioreanu A., Mot I.C., Horhat D.I., Balica N.C., Sarau C.A., Morar R., Domuta E.M., Dumitru C., Negrean R.A., Bumbu B.A. (2022). Development and Preliminary Characterization of Polyester-Urethane Microparticles Used in Curcumin Drug Delivery System for Oropharyngeal Cancer. Medicina.

[82] Lee S.Y., Wu S.C., Chen H., Tsai L.L., Tzeng J.J., Lin C.H., Lin Y.M. (2018). Synthesis and Characterization of Polycaprolactone-Based Polyurethanes for the Fabrication of Elastic Guided Bone Regeneration Membrane. BioMed Res. Int..

[83] Hu J.J., Liu C.C., Lin C.H., Tuan-Mu H.Y. (2021). Synthesis, Characterization, and Electrospinning of a Functionalizable, Polycaprolactone-Based Polyurethane for Soft Tissue Engineering. Polymers.

